# Anti-wrinkle effects of Seungma-Galgeun-Tang as evidenced by the inhibition of matrix metalloproteinase-I production and the promotion of type-1 procollagen synthesis

**DOI:** 10.1186/s12906-016-1095-z

**Published:** 2016-04-07

**Authors:** Min Kyoung Kim, Chae Young Bang, Gwang Jun Yun, Hyang-Yu Kim, Young Pyo Jang, Se Young Choung

**Affiliations:** Division of Pharmacognosy, College of Pharmacy, Kyung Hee University, Hoegi-dong, Dongdaemun-gu Seoul, 130-701 South Korea; Department of Preventive Pharmacy and Toxicology, College of Pharmacy, Kyung Hee University, Hoegi-dong, Dongdaemun-gu Seoul, 130-701 South Korea; Department of Life and Nanopharmaceutical Sciences, College of Pharmacy, Kyung Hee University, Hoegi-dong, Dongdaemun-gu Seoul, 130-701 South Korea

**Keywords:** Seungma-Galgeun-Tang, Anti-wrinkle, Matrix metalloproteinase-1, Type-1 procollagen

## Abstract

**Background:**

Seungma-Galgeun-Tang (SMGGT), a traditional herbal medicinal formula, has been used to treat various skin problems such as inflammation and rashes in Korean traditional medicine. In order to clarify the scientific evidence for the biological efficacy of SMGGT on the prevention of skin aging and in particular wrinkle formation, molecular anti-wrinkle parameters were evaluated in cultured human dermal fibroblasts.

**Methods:**

Standard SMGGT was prepared from KFDA-certified herbal medicines and the chemical fingerprint of SMGGT was verified by HPLC-ESI-MS to insure the quality of SMGGT. To evaluate the inhibitory effects of SMGGT on the synthesis of matrix metalloproteinase-1 (MMP-1) and type-1 procollagen, the content of MMP-1 and type-1 procollagen synthesizing enzymes in cultured human dermal fibroblasts were measured using an ELISA kit and Western Blot, respectively.

**Results:**

The treatment of SMGGT water extract significantly inhibited the production of MMP-1 and promoted type-1 procollagen synthesis concentration dependently.

**Conclusions:**

These results suggest that SMGGT has the potential to prevent wrinkle formation by down-regulating MMP-1 and up-regulating type-1 procollagen in human dermal fibroblasts.

## Background

Seungma-Galgeun-Tang (SMGGT) is a traditional herbal medicine in China and Korea that is prepared by decoction of four medicinal herbs including Cimicifugae Rhizoma, Puerariae Radix, Paeoniae Radix, and Glycyrrhizae Radix in water. This medicine has traditionally been used to treat epidemic diseases such as smallpox and measles, as well as urticaria by dispelling the superficial muscles, promoting eruption, tonifying the blood, and clearing away heat according to oriental medicinal theory and detoxification of inflammatory diseases [1]. Various studies have shown that SMGGT has anti-allergic effects [2], and inhibits cytopathic effects of human respiratory syncytial virus in cell lines from the human respiratory tract [3] and enterovirus 71 infection in a human foreskin fibroblast cell line [4]. Recently, a new clinical application for an old herbal preparation has been proposed by several Korean traditional clinics for prevention of wrinkle formation (unpublished clinical report).

Wrinkle formation is one of the primary characteristics of skin aging, and is a complex process that involves age-dependent decline of skin cell function. The major cause of winkles is loss of structural protein (type-1 collagen) in the dermal layer of the skin. Collagen makes up 70–80 % of the dry weight of skin and contributes to the stability and structural integrity of tissues. The progressive decline of type I collagen synthesis in the dermis contributes to the formation of wrinkles and aging skin [5]. Ultraviolet (UV) radiation is also known to induce skin damage, and chronic exposure has been shown to induce clinical and histological damage [6]. UVB was reported to induce transcription factors such as activator protein-1 (AP-1) and nuclear factor kappa-light-chain-enhancer of activated B cells (NF-κB) in the epidermis [7]. These factors impair the synthesis of collagen and induce the expression of matrix metaloproteinases (MMPs) [7–9]. Therefore, the evaluation of the inhibitory effects of specific materials on MMP-1 expression could be used to identify promising compounds that may inhibit the degradation of collagen [10–12]. Collagen directly influences skin tension, and maintenance of collagen structure is related to the intrinsic aging and photo-aging processes of the skin [13, 14].

In this study, we investigated the anti-wrinkle potency of SMGGT by evaluating its efficacy in reducing MMP-1 expression and promoting type-1 procollagen synthesis in human dermal fibroblasts.

## Methods

### Preparation of SMGGT water extract

Seungma-Galgeun-Tang (SMGGT) is composed of *Cimicifuga heracleifolia* Kom (Ranunculaceae, a root-like stem), *Pueraria lobata* Ohwi (Leguminosae, root), *Paeonia lactiflora* Pallas (Paeoniaceae, root), and *Glycyrrhiza uralensis* Fischer et DC (Leguminosae, root and stolon) which were mixed in order at the ratio of 2:1:1:1 and the total mixture weight was 125 g. KFDA-certified herbal medicines were purchased from a local herbal market in South Korea and their authenticity was re-confirmed by Professor Dong Il Kim, College of Korean Medicine, Dong-guk University, Ilsan, Korea. The dried plants were deposited as voucher specimen in the herbarium of the College of Korean Medicine, Dong-guk University (Ilsan, Korea) with voucher specimen numbers assigned as DUMCKM2015-044 (Cimicifugae Rhizoma), DUMCKM2015-001 (Puerariae Radix), DUMCKM2015-087 (Paeoniae Radix), and DUMCKM2015-014 (Glycyrrhizae Radix). SMGGT was extracted with distilled water at 100 **°**C for 4 h using a soxhlet extractor. The extract was passed through filter paper (Hyundai Micro Co., Ltd., Korea) and the filtrate was freeze-dried (yield 8 g) and stored at 4 **°**C.

### Chemicals and reagents

Acetonitrile (HPLC grade) and glacial acetic acid (99.0 % purity) were obtained from Duksan Pure Chemicals Co. (Ansan, South Korea). High purity nitrogen gas was provided by Shinyang Oxygen Co. (Seoul, South Korea). _L_-ascorbic acid was purchased from Sigma-Aldrich (St Louis, MO, USA). An MMP-1 immunoassay ELISA kit was purchased from Calbiochem Inc. (Darmstadt, Germany), and a type-1 procollagen immunoassay ELISA kit was purchased from Takara Bio Inc. (Otsu, Japan). Protease and phosphatase inhibitor cocktails were purchased from Roche (Mannheim, Germany). Western Blot was performed using following antibodies: anti-MMP-1 from Santa Cruz Biotechnology, Inc. (Santa Cruz, CA, USA), type-1 procollagen from Abnova Corporation (Taipei, Taiwan), and β-actin from Santa Cruz Biotechnology, Inc.

### HPLC-ESI-MS analysis

A total of 30 mg of SMGGT extract was dissolved in 1 mL of water and filtered through a 0.45 μm syringe filter (Millipore, Bedford, MA, USA) before being subjected to HPLC. The HPLC system consisted of a Dionex model P680 HPLC pump, ASI100 autosampler and UVD340U PDA detector operated by Dionex Chromeleon software. The Waters μBondapak C18 (Milford, MA, USA) column (300 × 3.9 mm i.d.; 5 μm) was selected for analysis. The UV/Vis detection wavelength was set to 315 nm. The mobile phase was comprised of acidified acetonitrile with acetic acid (0.1 %, solvent A) and acidified water with acetic acid (0.1 %, solvent B). All solvents were filtered through a 0.45 mm filter. The gradient program was 0 min, 2 % of solvent A; 120 min, 20 % of solvent A; 160 min, 100 % of solvent A at a flow rate of 0.8 mL per min. The injection volume was 10 μL.

An AccuTOF^®^ single-reflectron time-of-flight mass spectrometer equipped with an ESI source (Electrospray ionization, JEOL, Peabody, MA, USA) was operated with MassCenter system version 1.3.7b (JEOL). In the positive ion mode, typical values were set as follows: orifice 1 = 80 V and ring lens and orifice 2 = 10 and 5 V, respectively. The ion guide potential and detector voltage were set to 2000 V and 2300 V, respectively. ESI parameters were set as follows: needle electrode = 2000 V, desolvating chamber temperature = 250 **°**C, and orifice 1 temperature = 80 **°**C, respectively. Nitrogen gas was used as nebulizer and flow rate was 1 L/min, respectively. Nitrogen gas was also used as desolvating and flow rate was 3 L/min, respectively. Mass scale calibration was accomplished with a YOKUDELNA calibration kit (JEOL, Tokyo, Japan) for accurate mass measurements and calculations of the elemental composition. MS acquisition was set with a scan range of *m/z* 100 to 2000.

### Human skin fibroblast cell culture

Primary human foreskin dermal fibroblasts were established from biopsies of healthy male donor of 22 years old and in accordance with Institutional Review Board (IRB) approved by the Kyung Hee University Hospital (Seoul, Korea) (IRB approval no. 2012-01-006). The hospital obtained written informed consent from the donor, giving permission to collect their tissue and use for research purposes. The research adhered to the tenets of the Declaration of Helsinki. Fibroblasts were maintained in Dulbecco’s modified eagle medium (DMEM) supplemented with 10 % fetal bovine serum, 2 mM glutamine, penicillin (100 U/mL), and streptomycin (100 mg/mL) in a 37 **°**C humidified incubator containing 5 % CO_2_. The fibroblasts were cultured until 90 % confluency and then subcultivated. Cells cultured after five passages were used for the experiments.

### Cell viability assay

This assay measured the metabolic reduction of MTT to formazan (blue) by mitochondrial dehydrogenase, which is active only in living cells [15]. Human dermal fibroblast cells were pre-incubated in 24-well plates at a density of 10^4^ cells per well for 24 h. On the second day, cells were exposed to various concentrations (0, 100, 200, 400, and 500 μg/mL) of SMGGT water extract for 48 h. After 48 h, the media was removed and washed with phosphate-buffered saline (PBS, pH 7.4) and grown in 0.5 mg/mL MTT (prepared in PBS, filtered with a 0.2 mm membrane) at 37 **°**C. 4 h later, the MTT reagent was removed, formazan crystals were dissolved in dimethyl sulfoxide (DMSO) solution, and absorption values were read at 540 nm using an ELISA microplate reader (Bio-Tek instruments Inc., Winooski, VT, USA).

### UVB irradiation

The UVB light source was a sun lamp. To investigate the effects of UVB, human dermal fibroblasts cells were plated at a density of 10^4^ cells per well in a 24-well plate. The cells were cultured in DMEM media for 24 h. After 24 h, the media was replaced by 0.5 mL of phosphate-buffered saline (PBS pH 7.4), and the cells were subsequently exposed to UVB (40 mJ/cm^2^) light. Following irradiation, the cells were washed with PBS and cultured for 1 day in serum-free DMEM media with or without various concentration of SMGGT water extract (0, 100, 200, and 400 μg/mL) or _L_-ascorbic acid [16].

### MMP-1 inhibition assay in human dermal fibroblasts

The experimental group was divided into two, one is UVB-non-irradiated cells and the other is UVB-irradiated (40 mJ/cm^2^) cells. Human dermal fibroblasts (5 × 10^4^ cells) were pre-incubated in 24-well plates for 24 h. The cells were confluent and cultured with serum-free DMEM or serum-free DMEM containing various concentration (100, 200, and 400 μg/mL) of SMGGT water extract or _L_-ascorbic acid (200 μg/mL) for 48 h. For UVB-irradiated group, cells were washed with PBS and exposed to UVB (40 mJ/cm^2^) light. After irradiation, cells were incubated in serum-free DMEM. Cells in UVB-non-irradiated group were washed with PBS and further incubated in serum-free DMEM. After 24 h of incubation, the cell-free supernatants from those two groups were collected and used to assess MMP-1 degradation level. The effective inhibition of MMP-1 was evaluated using a colorimetric method (Abcam, Cambridge, MA, USA) by ELISA microplate reader [17]. _L_-Ascorbic acid was used as a positive control and the results were normalized with cell numbers that were confluent.

### Type-1 procollagen synthesis assay

Human dermal fibroblasts (5 × 10^4^ cells) were seeded onto 24-well plates for 24 h until they were confluent and then incubated with serum-free DMEM or serum-free DMEM containing various concentration (100, 200, and 400 μg/mL) of SMGGT or _L_-ascorbic acid (200 μg/mL) for 48 h. The study group was divided to UVB-non-irradiated and UVB-irradiated group. After incubation, the cell-free supernatants were collected from each well and the collagen contents were determined using a procollagen type-1 *C*-peptide assay kit (Takara Bio Inc., Otsu, Japan) with ELISA microplate reader. _L_-Ascorbic acid was used as a positive control and the results were normalized with cell numbers that were confluent.

### Western Blot analysis

Confluent cultured fibroblasts were pre-treated with various concentration of SMGGT water extract for 1 h and then exposed with UVB (40 mJ/cm^2^) light. After irradiation, cells were incubated with serum-free DMEM containing SMGGT and further incubated for 24 h. The cells were washed twice with cold PBS and lysed in lysis buffer (20 mM Tris–HCl (pH 7.4), 0.32 mM sucrose, protease inhibitor, 1 mM PMSF, 0.5 M EDTA (pH 8.0), 1 mM NaF, and 1 mM Na_3_VO_4_). Thirteen micrograms of protein per lane were separated by 8 % SDS-polyacrylamide gel electrophoresis. Proteins were transferred onto PVDF membranes in transfer buffer (25 mM Tris–HCl (pH 7.4), 192 mM glycine and 20 % v/v methanol). The transferred membranes were incubated for 2 h in blocking solution (5 % dried milk in Tris-buffered saline containing 0.1 % Tween-20) at room temperature. Blots were incubated with the appropriate primary antibodies at a dilution of 1:1000, and then further incubated with horseradish peroxidase conjugated secondary antibody at a dilution of 1:5000. Bound antibodies were detected using enhanced chemiluminescence plus kits (Amersham International, Little Chalfont, UK).

### Statistical analysis

Results are presented as means ± S.E.M. Statistically significant differences between groups were determined with one way analysis of variance (ANOVA) using statistical package for social sciences (SPSS) software. Multiple comparisons were performed using Turkey’s multiple-comparisons test. P-values <0.05 were considered to be statistically significant.

## Results

### Establishment of a standard HPLC fingerprint of SMGGT

**Fig. 1:**
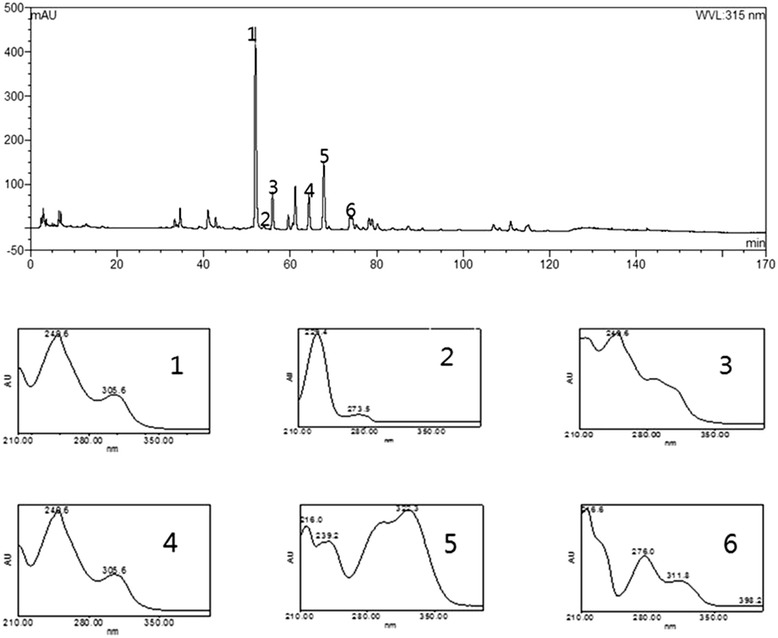
HPLC chromatogram of the SMGGT and UV-Visible absorption spectra of the six major peaks

There have been several reports on the HPLC profiles of single herbs included in SMGGT such as *Pueraria lobata* Ohwi and *Cimicifuga heracleifolia* Kom [18, 19], but there were no HPLC profiling studies to date that have looked at the SMGGT preparation. In order to establish a standard chromatogram for SMGGT, an HPLC study was performed. The representative chromatogram of SMGGT is shown in Fig. 1. Identification of major peaks on the chromatogram was accomplished using HPLC-ESI-MS.

### Identification of major components of SMGGT by HPLC-ESI-MS

The retention time, observed mass, mass difference, fragment ions, and proposed compounds of six peaks are listed in Table 1. The major components of SMGGT were identified by comparing both UV and MS spectra to their spectroscopic data in the literature [20–22]. The peak 1 and peak 4 showed identical protonated ion of *m/z* 417 in the positive ion mode but the fragment ion peaks were differentiated as the neutral loss of 120 Da in peak 1 (*m/z* 297, [M + H-C_4_H_9_O_4_]^+^) attributed to the characteristic cleavage of *C*-glycosides and the neutral loss of 162 Da in peak 4 (*m/z* 255, [M + H-C_6_H_10_O_5_]^+^) attributed to the characteristic cleavage of *O*-glycosides. Based on the molecular ions and their fragmentation patterns, peak 1 and peak 4 were identified as puerarin (daidzein-8-*C*-glucoside) and daidzin (daidzein-7-*O*-glucoside), respectively. Likewise, the peak 3 showed protonated ion at *m/z* 447 and the neutral loss of 120 Da same as in peak 1. Based on the retention time, MS spectrum, and 30 Da difference compare to the protonated puerarin ion at *m/z* 417, peak 3 was identified as 3’-methoxylpuerarin. The peak 2 represented protonated ion at *m/z* 481 and fragmented ion at *m/z* 179 due to the loss of single glucose and benzoic acid which specified it to be paeoniflorin. The peak 6 showed protonated ion at *m/z* 419 and fragmented ion at *m/z* 257 attributed to the loss of a glucose molecule and it was identified as liquiritin (liquiritigenin-4'-*O*-glucoside). Six major phytochemicals in SMGGT were identified as follows: isoferulic acid (from Cimicifugae Rhizoma), puerarin, 3'-methoxypuerarin, daidzin (from Puerariae Radix), paeoniflorin (from Paeoniae Radix), and liquiritin (from Glycyrrhizae Radix). The identities of KFDA- certified herbal medicines were reconfirmed by HPLC-ESI-MS to ensure the quality of the herbal medicines contained in SMGGT.

**Table 1:** The observed and calculated mass numbers of HPLC peaks of SMGGT

Peak No.	RT (min)	Theoretical mass [M + H]^+^	Observed Mass [M + H]^+^	Mass difference (mmu)	Fragment ions	Identification	Reference
1	51.93	417.11854	417.11338	−5.16	297.06931	puerarin	[21]
2	53.96	481.17096	481.17640	5.44	179.06642	paeoniflorin	[21]
3	55.91	447.12910	447.12316	−5.94	429.11301 327.07994	3'-methoxypuerarin	[20]
4	64.31	417.11854	417.11534	−3.20	255.05128	daidzin	[21]
5	67.74	195.06573	195.05935	−6.38	177.05273 149.05859	isoferulic acid	[22]
6	73.83	419.13416	419.13338	−0.78	257.07896	liquiritin	[21]

### Cell viability

**Fig. 2:**
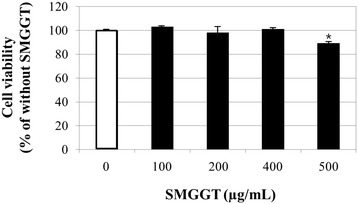
Cell viability test of SMGGT in human skin dermal fibroblast cells. Human dermal fibroblasts were cultured in DMEM until 80 % confluence. Cells were treated with various concentrations of SMGGT water extract for 48 h, after which the MTT assay was performed. Data are expressed as a percentage of the control (without SMGGT). Each evaluation was performed in triplicate

The cells were treated with various concentrations (0, 100, 200, 400, and 500 μg/mL) of SMGGT water extract and for 48 h. The SMGGT extract did not induce any cytotoxicity up to 400 μg/mL concentrations but showed slight reduction in cell viability (89.4 %) at 500 μg/mL (Fig. 2). The SMGGT water extract did not show any effects on cell proliferation (data not shown). Therefore, three different concentrations (100, 200 and 400 μg/mL) of SMGGT were used in this study.

### Inhibitory effects on MMP-1 production

**Fig. 3:**
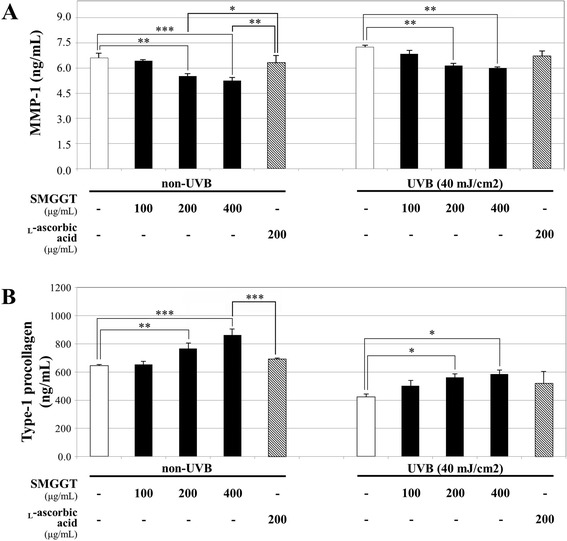
Effects of SMGGT on MMP-1 production (**a**) and type-1 procollagen synthesis (**b**) in UVB-non-irradiation or UVB-irradiation (40 mJ/cm^2^) group of human dermal fibroblasts. Cells were treated with various concentrations of SMGGT water extract or _L_-ascorbic acid for 48 h, and then cell supernatants were collected for ELISA kit. Statistical significance for the results were given as ^*^ *p* < 0.05; ^**^ *p* < 0.01 and ^***^ *p* < 0.001. _L_-Ascorbic acid was used as a positive control and the results were normalized with cell numbers that were confluent

To examine inhibitory effects of SMGGT on MMP-1, cells were cultured with SMGGT or _L_-ascorbic acid (positive control) for 48 h and then the cell-free supernatants were collected to quantify the level of MMP-1. The study was divided into two groups that non-UVB irradiated fibroblasts and UVB irradiated fibroblasts. The level of MMP-1 in vehicle-treated control from irradiated fibroblasts (7.25 ng/mL) was slightly increased compared with vehicle-treated control from non-irradiated fibroblasts (6.65 ng/mL) and both values were decreased dose dependently as treated SMGGT concentration increased (Fig. 3a). The non-UVB irradiated cells, SMGGT significantly decreased MMP-1 levels in a dose-dependent manner by 2.3 %, 16.3 %, and 20.5 % at concentrations of 100, 200, and 400 μg/mL percentile on the vehicle-treated group. The positive control, 200 μg/mL of _L_-ascorbic acid, decreased MMP-1 by 4.0 % which is similar to the 100 μg/mL of SMGGT. In a way, the UVB irradiated cells also dose-dependently decreased MMP-1 levels by 5.6 %, 15.2 %, and 17.1 % at concentrations of 100, 200, and 400 μg/mL, respectively (*p* < 0.001). The _L_-ascorbic acid decreased MMP-1 by 7.8 % similar to the 100 μg/mL of SMGGT.

### Promotion of type-1 procollagen synthesis

To evaluate the effects of SMGGT on type-1 procollagen synthesis, cells were cultured with SMGGT or _L_-ascorbic acid for 48 h and then the culture medium was collected. UVB irradiation significantly decreased the levels of newly synthesized type-1 procollagen compared with those of UVB-non-irradiated cells. As shown in Fig. 3b, SMGGT significantly increased type-1 procollagen synthesis in a dose-dependent manner by 2.2 %, 19.7 % and 34.8 % (percentile versus vehicle-treated group) at concentrations of 100, 200, and 400 μg/mL in UVB-non-irradiated groups, respectively (*p* < 0.001). The UVB-irradiated groups also showed a dose-dependent synthesis promotion of type-1 procollagen by the treatment with SMGGT; 19.7 %, 33.8 %, and 39.4 % increase at concentrations of 100, 200, and 400 μg/mL, respectively, and these effects were much potent as positive control, 200 μg/mL of _L_-ascorbic acid, which increased the level by 23.6 % versus vehicle-treated group. As a result, we concluded that the inhibition of MMP-1 production and promotion of type-1 procollagen synthesis by SMGGT were superior to the effects of _L_-ascorbic acid and these effects were more prominent when UVB was exposed. Similar pattern was observed with other herbal extract in previous report [23]. Therefore, we measured the changes of MMP-1 and type-1 procollagen expression by the treatment of SMGGT water extract in both UVB-non-irradiated and UVB-irradiated group to confirm the effects in molecular level.

### MMP-1 and Type-1 procollagen expression

**Fig. 4:**
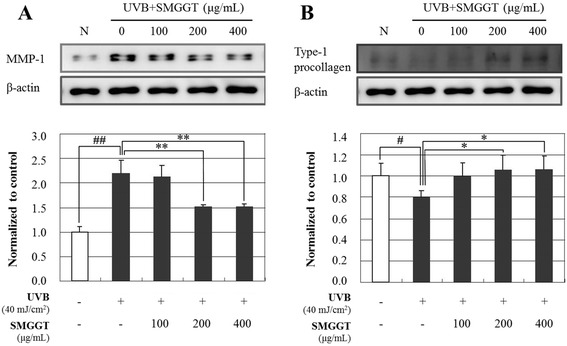
Effects of SMGGT water extract on UVB-induced expression of MMP-1 (**a**) and type-1 procollagen (**b**) in human skin dermal fibroblast cells. After UVB-irradiation (40 mJ/cm^2^), cells were cultured with or without various concentrations of SMGGT for 24 h. Each evaluation was performed in triplicate. Data are expressed as means ± S.E.M. ^#^ *p* < 0.05 and ^##^ *p* < 0.01 vs. vehicle control; ^*^ *p* < 0.05 and ^**^ *p* < 0.01 vs. UVB-irradiation only

To determine the anti-wrinkle effects of SMGGT water extract, the expression of MMP-1 (Fig. 4a) and type-1 procollagen (Fig. 4B) were evaluated in human dermal fibroblasts using Western Blot analysis. After UV-irradiation, the cells were incubated with 0, 100, 200, and 400 μg/mL of SMGGT for 1 day. As shown in Fig. 4a, SMGGT dramatically inhibited UVB-induced MMP-1 expression; the MMP-1 expression level decreased to 30.7 % at 200 μg/mL and 30.9 % at 400 μg/mL compared with the UVB-irradiation control group. Figure 4b showed that SMGGT significantly promoted the expression of Type-1 procollagen in UVB-irradiated human skin fibroblast cells in a dose-dependent manner; the type-1 procollagen expression level increased to 24.7 % at 100 μg/mL, 31.6 % at 200 μg/mL and 32.7 % at 400 μg/mL.

## Discussion

Seungma-Galgeun-Tang (SMGGT) is a traditional herbal medicine in China and Korea that is prepared by boiling four medicinal herbs, Cimicifugae Rhizoma, Puerariae Radix, Paeoniae Radix, and Glycyrrhizae Radix in water. This medicine has traditionally been used to treat epidemic diseases such as smallpox and measles, as well as urticaria by dispelling the superficial muscles, promoting eruption, tonifying the blood, and clearing away heat via detoxification in inflammatory diseases [1]. Through a recent survey on Korean traditional physicians, a new clinical application has been proposed for this preparation in several Korean traditional medicine hospitals as a preventative agent for excess wrinkle formation. Furthermore, Puerariae Radix was reported to increase mRNA expression for type-1 collagen in human osteoblast-like SaOS-2 cells [24] and Cimicifugae Rhizoma has been known to inhibit matrix proteinases on osteoarthritis [25]. The present study aimed to investigate the effects of SMGGT extract, which includes Puerariae Radix and Cimicifugae Rhizoma, on the anti-aging potential in human dermal fibroblasts and to provide molecular evidences for its anti-wrinkle efficacy in clinique.

The skin aging process can be classified into intrinsic aging, a natural course determined by internal genetic factors, and extrinsic aging, caused by external factors such as sun exposure, gravity, and smoking. Extrinsic aging caused by sunlight has been termed photo-aging [26]. UVB irradiation (290–320 nm) is the primary factor contributing to biological reactions in the skin, inducing inflammatory responses, apoptosis, and subsequent skin damage [27]. Interestingly, Seungma-Galgeun-Tang (SMGGT) had been reported to have anti-inflammatory effects in the skin [28]. In this study, we demonstrated the anti-aging activity of SMGGT water extract in human dermal fibroblast cells compared with those of _L_-ascorbic acid as a positive control. It has been reported that _L_-ascorbic acid, one of vitamin-C, is a potent anti-photoaging substance which is able to enhance collagen synthesis and inhibit MMP-1 expression [29]. UV irradiation induces photo-damage and causes premature skin aging [30]. Among the factors responsible for mediating UV-induced skin aging are matrix metalloproteinases (MMPs), which are up-regulated in dermal fibroblasts by UV irradiation [31]. UVB irradiation induces the expression of MMPs in human dermal fibroblasts which leads to the breakdown of collagen and other extracellular matrix proteins and causes pre-mature aging (photo-aging) of human skin [32]. Therefore, the inhibition of this increase in MMP expression has been reported to improve UV-induced photo-aging in terms of protection from collagen degradation [30]. As shown in Figs. 3 and 4, SMGGT water extract treatment significantly inhibited the production of MMP-1 and promoted the production of the type-1 procollagen in human dermal fibroblast cells, which provides strong support for the clinical efficacy for SMGGT. This was reconfirmed by the quantitation of MMP-1 and type-1 procollagen protein expression in UVB-irradiated human fibroblasts. The dose-dependent inhibition of MMP-1 expression and promotion of type-1 procollagen expression by the treatment with SMGGT demonstrates the clinical efficacy of this preparation for skin aging caused by extrinsic stresses including sunlight.

The molecular mechanism underlying the protective effects of SMGGT on UVB-irradiated skin aging were not revealed in this pilot study. Since collagen is known to be synthesized from dermal fibroblasts as precursor molecules called procollagen which is regulated by transforming growth factor *β*2 (TGF- *β*2) and activator protein-1 (AP-1), a transcription factor consisting of c-Jun and c-Fos promoting collagen breakdown by up regulating matrix metalloproteinases (MMPs) [33, 34], the potential molecular targets of SMGGT would be some of these important cytokines and transcription factor.

## Conclusions

In present study, a new application of the traditional herbal preparation SMGGT was tested by analyzing its effects on two major contributors to skin aging, MMP-1 and type-1 procollagen. The protective effect of SMGGT on UVB-irradiated skin aging in human fibroblast cells supports the clinical efficacy of SMGGT and suggests that this herbal preparation could be a potential candidate for an active ingredient in cosmeceutical products that prevent and cure wrinkle formation.

## Ethics approval and consent to participate

Primary human foreskin dermal fibroblasts were established from biopsies of healthy male donor of 22 years old and in accordance with Institutional Review Board (IRB) approved by the Kyung Hee University Hospital (Seoul, Korea) (IRB approval no. 2012-01-006). The hospital obtained written informed consent from the donor, giving permission to collect their tissue and use for research purposes. The research adhered to the tenets of the Declaration of Helsinki.

## Consent for publication

Not applicable.

## Availability of data and materials

The datasets supporting the conclusions of this article are included within the article.

## Abbreviations

ANOVA: one way analysis of variance
AP-1: activator protein-1
DMEM: Dulbecco’s modified eagle medium
DMSO: dimethyl sulfoxide
EDTA: ethylenediamine tetraacetate
ELISA: enzyme-linked immunosorbent assay
HPLC-ESI-MS: high performance liquid chromatography-electro spray ionization-mass spectrometry
KFDA: Korea Food and Drug Administration
MMP-1: matrix metalloproteinase-1
MTT: 3-(4,5-dimethylthiazol-2-yl)-2,5-diphenyltetrazolium bromide
NF-κB: nuclear factor kappa-light-chain-enhancer of activated B cells
PMSF: phenylmethanesulfonyl fluoride
SMGGT: Seungma-Galgeun-Tang
SPSS: statistical package for social sciences
UVB: ultraviolet light B
